# Dynamic contextual influences on social motivation and behavior in schizophrenia: a case-control network analysis

**DOI:** 10.1038/s41537-021-00189-6

**Published:** 2021-12-09

**Authors:** Varsha D. Badal, Emma M. Parrish, Jason L. Holden, Colin A. Depp, Eric Granholm

**Affiliations:** 1 Department of Psychiatry, University of California San Diego, San Diego, CA USA; 2 Sam and Rose Stein Institute for Research on Aging, University of California San Diego, San Diego, CA USA; 3 San Diego State University/University of California San Diego Joint Doctoral Program in Clinical Psychology, San Diego, CA USA; 4 VA San Diego Healthcare System, La Jolla, CA USA

**Keywords:** Schizophrenia, Human behaviour

## Abstract

Contextual influences on social behavior and affective dynamics are not well understood in schizophrenia. We examined the role of social context on emotions, and the motivation to interact in the future, using dynamic network analysis of ecological momentary assessment (EMA) data. Participants included 105 outpatients with schizophrenia or schizoaffective disorder (SZ) and 76 healthy comparators (HC) who completed 7 days, 7 times a day of EMA. Dynamic networks were constructed using EMA data to visualize causal interactions between emotional states, motivation, and context (e.g., location, social interactions). Models were extended to include the type and frequency of interactions and the motivation to interact in the near future. Results indicated SZ networks were generally similar to HC but that contextual influences on emotion and social motivation were more evident in SZ. Further, feedback loops in HC were likely adaptive (e.g., positive emotions leading to social motivation), but most were likely maladaptive in SZ (e.g., sadness leading to reduced happiness leading to increased sadness). Overall, these findings indicate that network analyses may be useful in specifying emotion regulation problems in SZ and that instability related to contextual influences may be a central aspect of aberrant regulation.

## Introduction

Social dysfunction is common in schizophrenia and its determinants are complex^1–3^. Intensive longitudinal data on social behavior gathered through Ecological Momentary Assessment (EMA) have begun to expand understanding of the dynamic links between symptoms, motivation, and social behavior. This work has allowed anxiety to be viewed as reduced anticipation of positive outcomes of social interactions, in turn leading to reduced social participation^4,5^. Assumptions about the foundational influences on social behavior have also been challenged with EMA, as the link between social capacity and social behavior as measured by EMA are not consistently observed^6^. Therefore, EMA provides a window into social dynamics in schizophrenia that may expand understanding of the mechanism underlying social dysfunction.

A less explored capability of EMA in assessing social behavior is the ability to assess contextual variability in social interactions^7^ (those that are associated with some degree of intimacy, e.g., friends, family, coworkers, vs. non-social refer to those with staff and treatment providers, see also ref. ^8^). Research has primarily focused on factors intrinsic to the individual in evaluating the affective experience of interactions (e.g., motivation, symptoms), with less attention to outer behavioral context, such as the influence of location (e.g., at home, out of the home) or variation in interaction partners, which may also influence social motivation and behavior. There is some evidence to suggest that contextual variation is important. While a review^9^ and an EMA study^10^ of SMI by the same group did not find significant differences between patients and healthy controls on time spent alone or involvement in interactions, the included studies did not fully examine who was interacting with the patient. We^8^ found a double dissociation whereby controls reported significantly more reciprocal social interactions (e.g., with family, friends, coworkers), but significantly fewer instrumental or non-social interactions (e.g., with treatment providers, board-and-care staff) than patients with SMI, so the social and non-social instrumental interactions summed to a similar amount of total interactions for both groups. Moreover, social, but not non-social, interactions were correlated with in-lab measures of negative symptoms and social functioning. Thus, impairments were detected only when parsing the social context into meaningful reciprocal social interactions. In related EMA work on the context of being at home versus being outside of the home, people with schizophrenia experienced greater anxiety out of the home than healthy comparators, whereas healthy comparators experienced greater positive affect outside of the home^5^. As such, social motivation may depend upon where one is (home or away), who one is with, and variability in affective experiences across these different contexts^10,11^.

Since interactions between positive and negative affect, social motivation, and social behavior are complex, likely bi-directional, and context-dependent, associations are difficult to explore with bivariate analyses. Network models are based on time series analysis and account for internal structures in the data set such as auto-correlations, trends between variables, and feedback loops, to obtain a better understanding of forces and structures that might have produced the data. Traditional statistical methods usually provide a more static view, not much beyond associations between a few variables. In respect to this set of analyses, we focused on context as a modifier of affective experiences, which was identified in prior work but only at the bivariate level. Network analysis is a statistical tool that is well suited to EMA data, in that it identifies correlations between variables observed repeatedly over time and their temporal sequences. In network analysis, relationships are represented as weighted connections, or edges, between EMA variables as reflected in a network graph. Networks provide a dynamic view of multiple variables enabling qualitative comparison between groupings of interest, well as quantitative assessments of the density of networks (how connected each node is with other nodes) and the presence of feedback loops (variables that lead to increases or decreases in other variables in time-lagged analyses). Network analysis has been applied in mental health research analyzing cross-sectional data^12^, time-series analysis^13^, and EMA data^13,14^. Network analyses provide a natural fit with EMA data where context, social behavior, social motivation, and affective experience are sampled repeatedly over time.

In this secondary analysis of a previously reported EMA data set^8^, also analyzed^5^, we explored network dynamics of social behavior, affect, and social motivation in schizophrenia in a sample of 105 outpatients with schizophrenia and 76 healthy comparators. This study allows us to examine other concepts (feedback loops and density) using causal network analysis while also breaking it down into the specific types of relationships and adding to the examination of social motivation.

Participants completed 7 EMA surveys a day for 7 days. We applied network models to evaluate relationships between positive and negative affective experience and social motivation in three social contexts (a) being at home vs. out of the home, (b) being alone vs. with others, and (c) types of recent interactions partners (e.g., family, friends, strangers). In these networks, we predicted that context factors (being alone, at home) would have stronger connectivity to other nodes in network models of people with schizophrenia than healthy comparators, particularly in respect to negative affect based on our prior work^8^ and the follow up^5^. Connections between positive affect and motivation may be stronger in networks of healthy comparators. Finally, we explored the density of networks and feedback loops in graphs, which are sequences of lagged associations, and qualitatively evaluated feedback loops in each group along the dimensions of adaptivity (i.e., leading to increases in positive emotions or motivation and/or reduction in negative emotions) or likely maladaptivity (i.e., the converse: decreases in positive emotion or motivation and/or increase in negative emotions), and the influence of context.

## Results

### Sample characteristics

The sample consisted of 181 participants (67.9% men, 41.9% Caucasian, 34.2% African American, 13.2% Hispanic), aged 20–65 years (*M* = 50.6, SD = 10.6) at the time of visit split into healthy control (HC) (*n* = 76) and people with schizophrenia (SZ; *n* = 105) (Table 1). Our original study^8^ defined inadequate adherence as <33% of the surveys being completed. Overall, 85% of the surveys were completed, the statistics for incomplete and excluded surveys was not significant across the groups (HC = 6.6% [5/76], SZ = 2.9% [3/103]; *χ*^2^(1) = 1.38, *p* = 0.241)^8^. Additionally, 6 of the phones were lost or malfunctioned and resulted in complete EMA data loss^8^. Mean, standard deviation, effect sizes, and significance (across groups) for all variables are also presented in Table 1. The HC and SZ groups significantly differed from each other in mood (i.e., happy, sad, anxious, relaxed), location, and social motivation variables. Consistent with prior research, the HC group spent significantly less proportion of their sampled time at home compared to SZ (54.7% vs. 72.1%, Cohen’s *d* = −2.06, *p* < 0.01). The amount of interaction with friends was very similar (Cohen’s *d* = −0.03, *p* = 0.11), however participants with SZ interacted less with family (Cohen’s *d* = 0.21, *p* < 0.01), coworkers (Cohen’s *d* = 0.16, *p* < 0.01), and others (Cohen’s *d* = 0.09, *p* < 0.01), compared to HC. People with SZ interacted with roommates (Cohen’s *d* = −0.28, *p* < .01), staff (Cohen’s *d* = −0.23, *p* < 0.01) and treatment providers (Cohen’s *d* = −0.12, *p* < 0.01) at rates about twice as much or more by proportion when compared to HC.

**Table 1:** Clinical characteristics of the study sample (*n* = 181).

	HC (*n* = 76)^a^	SZ (*n* = 105)^b^	Cohen’s *d*	Student-*t* or *χ*^2^	*p*-val
Sex (% female)	36.8%	28.8%	–	1.29	0.257
Age, *M* (SD), range	49.2 (11.9), 22.2–65.4	51.9 (9.2), 26.4–65.0	−0.74	−1.69	0.094
Education (years), *M* (SD), range	14.61 (1.8), 12–18	13.0 (1.90), 8–20	1.15	5.6	<0.001
Race (%)			–	9.8	0.135
Caucasian	50.0	37.5			
African American	22.4	41.3			
Hispanic/Latino	15.8	11.5			
Other	11.8	9.6			
Marital status (% married)	22.4	4.8			
Employment status (% employed)	56.6	16.5		33.3	<0.001
Interaction types (EMA records containing type) *N* (%)			–	604.3	<0.001
Alone	887 (23.8%)	1457 (26.1%)			
Roommate	315 (8.5%)	953 (17.1%)			
Family	756 (20.3%)	632 (11.3%)			
Treatment provider	79 (2.1%)	274 (4.9%)			
Friends	706 (19.0%)	1021 (18.3%)			
Coworker	321 (8.6%)	182 (3.3%)			
Staff	43 (1.2%)	397 (7.1%)			
Other	613 (16.5%)	663 (11.9%)			
Interaction types (total per individual) *M* (SD)					
Alone	12.5 (9.4)	14.1 (10.9)			
Roommate	8.5 (8.2)	11.6 (11.0)			
Family	14.8 (13.0)	9.9 (10.1)			
Treatment provider	2.6 (2.4)	4.0 (3.8)			
Friends	10.9 (7.7)	10.6 (9.1)			
Coworker	8.2 (8.3)	4.4 (4.3)			
Staff	3.9 (4.2)	7.4 (8.8)			
Other	8.9 (6.5)	7.1 (6.6)			
Interaction category, *M* (SD)					
Social	0.4 (0.6)	0.3 (0.5)	0.10	5.9	<0.001
Non-social	0.4 (0.6)	0.5 (0.7)	−0.14	−7.9	<0.001
Clinical measures					
BPRS-positive symptoms	–	9.3 (3.8)	–	–	–
CAINS motivation and pleasure	–	15.9 (6.4)	–	–	–
CAINS expression	–	3.5 (3.3)	–	–	–
EMA variables, *M* (SD)					
Happy	5.4 (1.3)	4.7 (1.7)	0.61	21.7	<0.001
Sad	1.6 (1.1)	2.6 (1.8)	−0.81	−29.4	<0.001
Anxious	1.5 (1.1)	2.5 (1.7)	−0.80	−29.4	<0.001
Relax	5.3 (1.5)	4.7 (1.7)	0.49	16.7	<0.001
At home	32.8 (28.0)	43.3 (24.4)	−2.06	−16.5	<0.001
Interaction count	1.9 (1.9)	1.8 (1.9)	0.06	1.8	0.07
Social motivation	5.3 (1.8)	4.3 (2.2)	0.67	20.6	<0.001

*BPRS* Brief Psychiatric Rating Scale, *CAINS* Clinical Assessment Interview for Negative Symptoms.

^a^Some data unavailable for 1 subject, ignored.

^b^Some data unavailable for 3 subjects, ignored.

#### Affect and social motivation

**Fig. 1:**
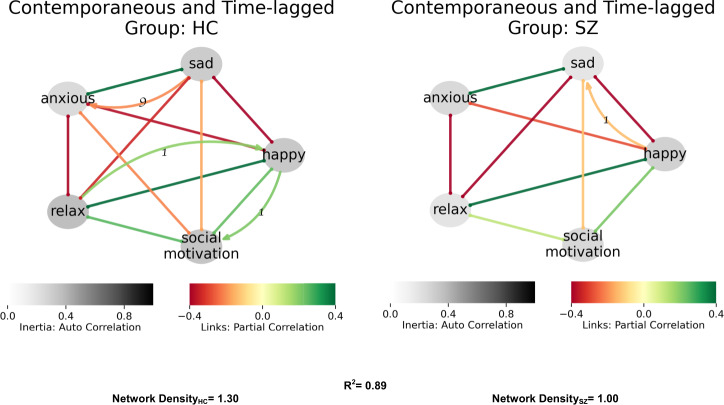
Affect and motivation. HC network is denser, showing greater connectivity between positive affect and motivation. Happy, relax, and social motivation shows the likely adaptive feedback loop that sustains motivation. The red line represents negative associations while the green lines represent positive associations. Color-depth of links represents the strength of associations (deeper means stronger). Curved lines represent lagged associations and non-curved contemporaneous.

Networks evaluating affect and social motivation in SZ and HC, displayed in Fig. 1, were similar to one another (*R*^2^ = 0.89). SZ networks were less dense, or connected, indicating mood had less impact on motivation than for HC (1.0 vs. 1.3). In HCs, contemporaneous links were evident for each affect, and a time-lagged association was observed between happiness and subsequent motivation. In contrast, in SZ, there were no time-lagged associations and there was no link between positive affect and subsequent social motivation. Feedback loops shown in HC, but not SZ, (loops L1, L2, L3 in Table 2) were each in likely adaptive directions (e.g., greater happiness leading to greater social motivation) and prolonged sadness reducing anxiety (loop L4 in Table 2). In contrast, the only feedback loop in SZ was likely maladaptive, with sadness reducing subsequent happiness (loop L5 in Table 2). Thus, HC networks with affect and motivation indicated a higher density network and likely adaptive feedback loops, whereas SZ networks had likely maladaptive feedback loops. (Fig. 1).

**Table 2:** Summary of feedback loops in network models.

Loop	Figure	Group	Variables	Feedback type	Adaptive	Includes context
L1	1	HC	↑Relax -> ↑Happy -> ↑Social motivation -> ↑Relax	Positive	Adaptive	No
L2	1, 2a	HC	↑Happy -> ↑Relax -> ↑Happy	Positive	Adaptive	No
L3	1, 2b	HC	↑Social motivation -> ↑Happy -> ↑Social motivation	Positive	Adaptive	No
L4	1, 2a	HC	↑Anxious -> ↑Sad -> ↓Anxious	Negative	Adaptive	No
L5	1	SZ	↑Sad -> ↓Happy -> ↑Sad	Positive	Maladaptive	No
L6	2a	SZ	↓Relax -> ↑Anxious -> ↓At home -> ↓Relax	Positive	Maladaptive	Yes
L7	2a, 2b	SZ	↑Alone -> ↑At home -> ↑Alone	Positive	Maladaptive	Yes
L8	2b	SZ	↑At Home -> ↑Relax -> ↓Anxious -> ↑At home	Positive	Coping strategy	Yes
L9	3a	SZ	↑Friends -> ↑Happy -> ↓Friends	Negative	Maladaptive	Yes

Identified loops were unique to and significant only for the group indicated.

#### Affect and social context

**Fig. 2:**
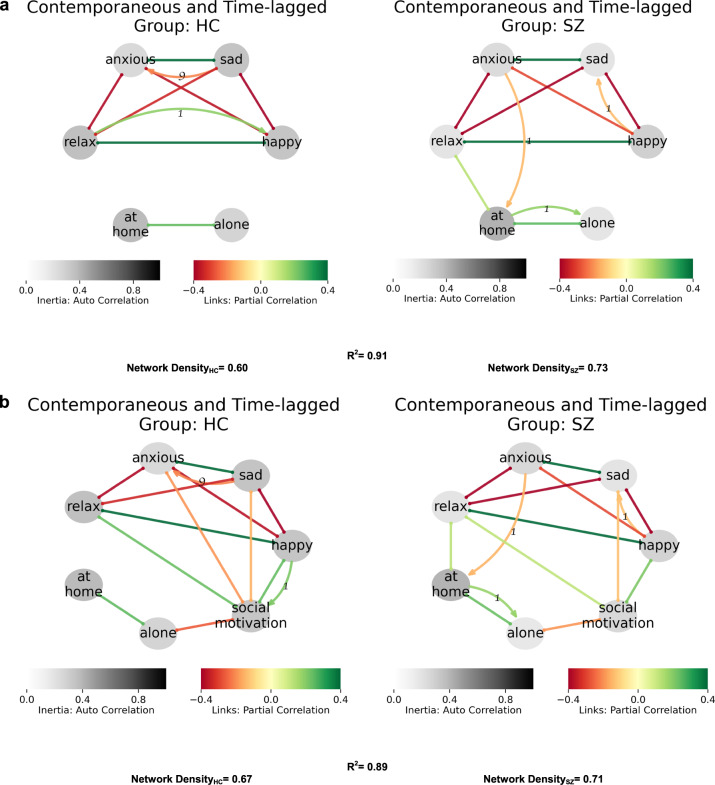
Context, affect, and social motivation. **a** SZ networks show greater densities and affect subgraphs connected to context. Likely maladaptive feedback loops are evident in SZ networks, with lagged associations suggesting greater anxiety in anticipation of leaving home and being at home increases future time alone. **b** Social motivation has a self-sustained positive-feedback loop with a happy mood in HC. Not only such a loop is lacking in SZ, the associations with happy and relax states are weaker.

Figure 2a shows the causal networks for affect and social context (being alone, at home). The networks for SZ displayed greater influence of context on mood, which resulted in higher network density. In contrast, no social context links were evident in HC. In terms of feedback loops, being at home was positively linked with relaxation and negatively linked with anxiety in SZ, but not HC (loop L6 in Table 2). Moreover, there was a likely maladaptive, self-reinforcing positive-feedback loop in SZ, whereby being at home increased being alone which in turn was contemporaneous with and increased being at home in the future (loop L7 in Table 2).

#### Affect, social context, and motivation

Finally, Fig. 2b combines affect, context and motivation. Networks between HC and SZ were similar, as goodness of fit was high (*R*^2^ = 0.89). However, as with the models with context above, the social context (alone, at home) network was denser for SZ relative to HC participants (0.71 vs. 0.67). The presence of feedback loops between motivation and positive affect (L3 in HC), being at home, and subsequent time alone (L7 in SZ) with lagged associations suggesting greater anxiety in anticipation of leaving home (L6, L8 in SZ) and being at home increases future time alone (L7 in SZ) were seen in multiple networks, suggesting these are invariant features (Table 2). In summary, when context (alone, at home) was included in network analysis, affect was closely linked to social context among SZ.

#### Interaction partners and affect

**Fig. 3:**
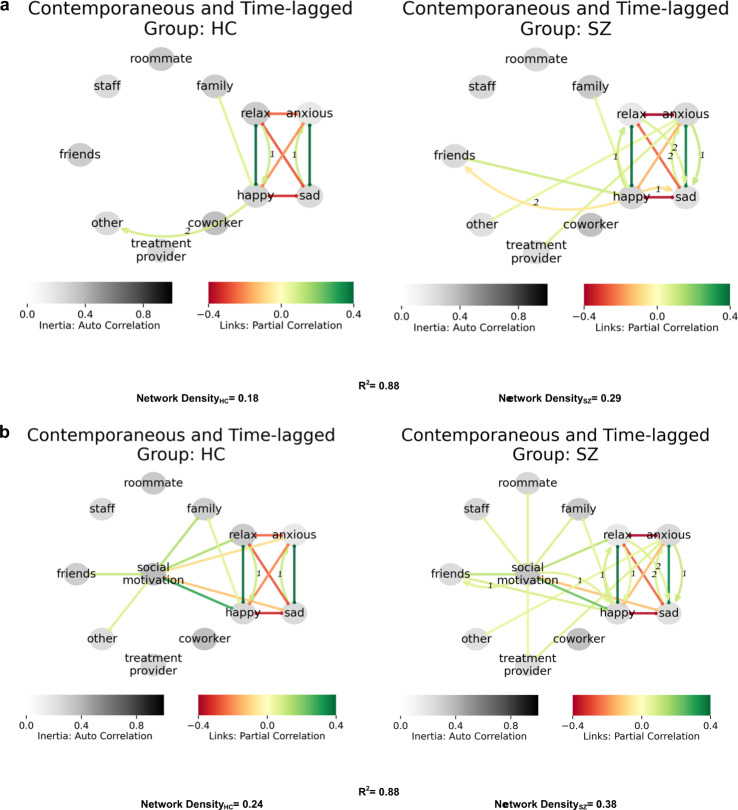
Interaction partners, affect, and motivation. **a** SZ networks of interaction partners, and affect were denser in the SZ group compared to HC. Link between interaction with others and happy is lacking in SZ, while strong link to anxiousness is evident. **b** Social motivation translates to interaction with other in HC but not SZ. Further, social motivation in SZ is highly connected (node centrality) and hence an excellent suggested candidate for intervention. Treatment providers are often associated with both motivation and anxiety in SZ.

We next evaluated networks for affect and specific interaction partners, across HC and SZ groups (Fig. 3a). The networks show strong goodness of fit (*R*^2^ = 0.88), yet the networks were denser for SZ (0.18 vs. 0.29). For HC, there was a contemporaneous positive association between family interactions with happiness and a positive lagged association between happiness and future interactions with “others”. In SZ, similar contemporaneous positive associations were observed between family (and friends) with happiness, but happiness did not predict future interactions. Rather, in the SZ networks anxiety was positively associated with interactions with treatment providers and with “others”. Although the positive contemporaneous association between happiness and friend interactions was intact in SZ, the additional negative lagged link with happiness predicted reduced subsequent interactions with friends (loop L9 in Table 2). Higher density of the SZ network suggests a greater impact of interaction partners context on affect (or vice versa)

#### Interaction partners, affect, and motivation

The combined networks for specific interaction partners, affect, and social motivation across HC and SZ groups (Fig. 3b) were again denser for SZ compared to HC (0.38 vs 0.24). Family and friends had similar contemporaneous relationships with positive affect and social motivation for SZ and HC, and social motivation was related to most relationships in SZ, except “others” which was related to social motivation in HC. In SZ, anxiety was linked with “others” and treatment providers. In addition, a lagged relationship emerged whereby positive affect during interactions with friends was associated with increased motivation for and participation in future interactions with friends in SZ (Fig. 3b).

In summary, the networks for HC and SZ preserved most of their respective connectivity discussed (in Interaction Partners and Affect section and Fig. 3a), with the key exception of the lagged negative link between happiness and future interaction with friends in the SZ network (loop L9 in Table 2). However, the introduction of motivation into the affect and interaction partner network eliminated the lagged negative link between happiness and future interaction with friends, suggesting the link is conditionally dependent on motivation. Finally, the networks were denser for SZ compared to HC, indicating greater contextual influence in SZ.

## Discussion

There is increasing evidence of emotion–behavior decoupling in SZ (i.e., impairment in translating emotion to motivated behavior); one study found it was most pronounced in people with SZ (and schizoaffective) among the representative clinical groups^15^. Our findings that affect and motivation were more de-coupled in SZ are consistent these emotion–behavior studies^16,17^. In this study, we evaluated the influence of several social contexts on affect and social motivation in schizophrenia via network analyses of intensive longitudinal data gathered through EMA.

While network analyses are inherently exploratory and require replication, several potentially important distinctions between SZ and HC emerged. In models without context, including only affect and motivation, HC networks were more dense than in SZ with weaker lagged associations between affect and motivation in SZ. Our findings are in agreement with a previous study^18^ by our group in a different sample and EMA protocol where positive affect was a strong predictor of greater social interaction. In contrast, models that included context revealed greater density in SZ, with more connections between being alone, location, type of interaction partner, and affect and social motivation. Thus, context had greater impact on SZ. In addition, feedback loops in HC were generally in the direction of likely adaptive states that were independent from context (e.g., happiness leading to greater relaxation), whereas the great majority of networks were likely maladaptive in SZ (e.g., sadness leading to diminished happiness; time at home leading to greater time at home), which is suggestive of negative affect persistence in SZ, consistent with prior literature^19–22^. Moreover, interactions with strangers in SZ were more negatively experienced than in HC. Taken together, these findings indicate that network models may be useful in accounting for complex dynamics of social motivation and emotions in social behavior, and evidence greater influence of context on affect and motivation in SZ.

Our findings are consistent with broader findings of emotion regulation deficits observed in SZ^23,24^, as SZ networks did not show the likely adaptive feedback loops found in HC linking positive affect (happy, relaxed) with more sustained positive mood, and negative feedback loops among negative affect variables (sad, nervous) were not evident in SZ. Moreover, our findings parallel the limited prior EMA network analysis research indicating generally higher network density in SZ^14^, with the key exception of our lower-density affect-motivation network that did not include a context (Fig. 1).

To our knowledge, this increased influence of context is a pertinent finding, and suggests that context has a greater influence on emotion and social motivation in SZ than HC. One possible mechanism is that increased contextual influence is linked to diminished anticipatory pleasure deficits in schizophrenia^25^, such that in the absence of intrinsically driven (e.g., anticipation) influences on subsequent experiences contextual influences may produce a greater role^26^. The network models reported here identified a link between happiness and subsequent social motivation in HC, whereas no such feedback loop was evident in SZ. A second mechanism (which is not mutually exclusive) may be that differences in the social context itself account for the variation in networks across SZ and HC. In particular, people with schizophrenia are more likely to reside more controlled or structured living environments, wherein context changes (e.g., leaving the home) may be less under individual control. In addition, they may also be more likely to live in adverse or deprived contexts (e.g., less safe neighborhoods), which could account for greater influence of context on mood and affect^27,28^. In this study, we did not investigate the quality of the interactions or estimate adverse experiences, but studies on environmental influences such as neighborhood quality or social network characteristics could be integrated with EMA and network analyses^8^.

Additionally, HC and SZ had unique affective linkages with the “other” category of partners, which may include interactions with strangers or unknown people. People with schizophrenia experienced greater nervousness, whereas healthy comparators had positive links between interactions with “others” and social motivation. Similarly, people with schizophrenia experienced nervousness in treatment provider interactions. Taken together, this suggests that people with schizophrenia experience negative emotions and limited positive emotions when interacting with unfamiliar social partners. It is unclear if these negative experiences are a result of psychotic symptoms (e.g., paranoia) and/or social anxiety^29,30^, the latter highly common yet poorly understood aspect of schizophrenia^10,31^. Again, however, it may be that the unique context of interactions and circumstances in which people with schizophrenia may meet strangers may be less reinforcing and stigmatizing. In terms of clinical implications, it may be that interventions that reduce barriers to the frequency of contact with existing social connections and provide exposure to alter the affective experience of stranger contacts such as with cognitive-behavioral therapy (CBT) or social cognition training could be beneficial. For example, the network models (Fig. 3) suggested that friendships were more rewarding in SZ and associated with increased motivation for and subsequent future participation in interactions with friends.

There are several limitations to this study. First, network analyses require replication, and our sample size was insufficient to create test and validation samples. As such, these findings are tentative. Secondly, the evaluation of networks is somewhat qualitative and descriptive in nature, and methods for quantitative comparison of networks analyses with EMA data are still being developed. It is important to note that the *R*^2^ values for networks indicated that SZ and HC samples are similar (as most network edges are conserved across the groups), hence the differences in structure and density should not be overstated. Third, the sample is comprised of middle-aged, predominantly male stable outpatients and may not apply to other patient populations, and we also lacked sample size to evaluate differences within patient subgroups in network properties that may impact the networks (e.g., living independently vs. in a supported setting). Fourth, participants were only sampled for one week, and while variables were stationary (without trends), these data may not be reflective of typical affect and behavior patterns. Relatedly, we did not account in our models for night-to-day lags and so lags are modeled as if continuous. Fifth, although the inter-relationships between variables hint at differences in processes related to the regulation of emotions, more direct measures of emotion regulation administered through EMA^32,33^ would certainly deepen the ability to understand contextual influences on affect and motivation. Finally, it is worth noting that paranoia severity is an important factor in social-threat perception^34^ (and hence in avoidance behavior), analyses of affect and social motivation provide an incomplete picture. Bivariate analysis in our sample revealed that average motivation reported by an SZ individual to interact in future was significantly and negatively related to CAINS total score at baseline (Spearman’s *r* = −0.32, *p* < 0.001), however, the relationship with BPRS-positive scale was not significant.

In terms of the next steps, social sensing may provide greater detail on the conjoint relationships between affect, motivation, and social context. This would include a naturalistic study of the process of interactions, from initiation to engagement. It would be particularly useful to understand the influence of social structure and these interaction networks on the emergence of symptoms over time. Perhaps contextual reactivity may accompany increases in symptoms and within-person changes in social behavior from one’s average interaction habits may be evaluated with changes in network relationships over time. It would also be useful to understand the influence of the broader environment, e.g., living alone or with others, opportunities for interactions on these networks, and how these influences could be improved to enhance the emotional experience and social motivation in SZ. We also hope to include paranoia severity in our EMA models in future studies.

## Method

### Participants

These are secondary analyses of data from an observational study aimed at evaluating functioning with ecological momentary assessment, and the study is available here^8^. Participants were included in the study if they: (1) met criteria for schizophrenia or schizoaffective disorder on the Structured Clinical Interview for DSM-5^35^ or, for healthy controls had no current or past mood, anxiety, or psychotic disorder; (2) were ages 18–65; (3) were fluent in English, and (4) could provide informed consent. Participants were excluded if they had experienced a head trauma with loss of consciousness, a seizure disorder, cerebrovascular accident or dementia, or a current diagnosis of substance use disorder that met DSM-5 criteria in the past year.

The study complied with all relevant ethical regulations for work with human participants and was approved by the San Diego Veterans Affairs (VA) IRB. Further, all participants provided written informed consent.

### Procedure

First, participants completed in-lab assessments, and were given a Samsung Android OS smartphone. EMA surveys on the smartphone began the following day via Samplex software. Participants were surveyed seven times a day for seven days. The surveys were given at a stratified random interval in 1.5-h windows from 9 am to 9 pm. After receiving a signal, participants had 15 min to respond, and were paid $1 for every survey completed. After seven days of EMA surveys, participants returned to the lab, returned the smartphone, and were given payment.

### EMA measures

In measuring affect, all participants rated how happy, sad, relaxed, and nervous they were on Likert scales ranging from one (not at all) to seven (extremely). Participants reported much time they spent at home in the past hour and could indicate if they were home from 0 to 60 min (entirely away or entirely at home) in 15-minute increments. They were also asked how many interactions they had over the past hour (from 0–None to 6 or more). Participants who indicated that they had an interaction in the past hour reported whether they interacted with “family,” “roommates or fellow residents,” “staff where I live,” “friends or acquaintances,” “coworkers or classmates,” “treatment providers (case manager, counselor, doctor, etc.),” or “other.” Participants also indicated how much motivation they had for engaging in future interactions (one, “not at all” to seven, “very much”). Each type of partner is analyzed separately. Although we did not discern between virtual or in-person interactions in the analyses, participants were coached in terms of interactions being defined as both in-person and on phones/devices. Additionally, the EMA survey displayed an informational screen stating the same when participants had questions about the interactions. Further, they were provided an opportunity to ask questions after being provided a sample survey. We refer interested readers to more detailed description of the EMA items used in this study^8^.

### Data analysis

We first calculated descriptive statistics and compared the people with schizophrenia with healthy comparators along EMA-derived variables. We then created network models for the following variable clusters with each group: (a) affect and motivation, (b) affect, motivation and context, (c) interaction partners and affect and (d) interaction partners, affect and motivation. The Python Tigramite package was used to establish temporal networks. The package calculates a partial correlation between variables and used momentary conditional independence to eliminate false-positive associations, including both cross- and auto-regressive associations in order to estimate both contemporaneously and lagged networks^36^.

In data processing, lags were restricted to a two-day window [*t*_max_ = 0 for contemporaneous, *t*_max_ = 14, or 2 days] elapsed or real time, irrespective of adherence. Values that were specified very sparsely (such as “how enjoyable was the interaction”), were assigned midscale (4 on scale 1–7) to provide continuity in the time series analysis. Time series are constructed by concatenating observations of all subjects in groups of interest (HC, SZ) preserving relative order (in splicing together of time series, the corresponding observations are ordinally consistent). Only significant associations at the *p* < 0.01 are reported. We limited the number of variable per analysis to 10 based on ref. ^37^ except out of necessity when analyzing interaction partners. To ensure stationarity, or that the variables are relatively stable over time and without trends, we used the Augmented Dickey–Fuller unit root test. The test indicated all variables to be stationary over time (*p* < 0.01). Overall, 9.16% of values across all variables relevant to the study (affect, context, people, and motivation) were missing. The algorithm can handle missing data by ignoring missing values while consistently handling time lags to a certain extent^37^. Further, when the interaction counts were blank for various types of people, they were interpreted as zero for the type, as designed. The algorithm implementation generates an error when data is insufficient and correspondences between variables cannot be established, however, we did not encounter errors of this type.

The networks were built incrementally, expanding the set of variables examined at each stage. Smaller networks (fewer variables) are easier to interpret. Causal networks are not always structurally additive. The network analysis algorithm eliminates conditionally dependent edges (A large network with confounding variables will have redundant edges unless conditional independence is tested, e.g., if A is related to B and B to C, then A is also related to C, however, conditional independence test would eliminate the last link). The combined network retains edges describing distinct processes (without overlap) while removing edges that are conditionally dependent on other edges^36^.

Networks were evaluated on several dimensions. First, we calculated *R*^2^, a goodness of fit measure between networks, in this case, SZ and HC. *R*^2^ provides a normalized measure of similarity by subtracting the sum of squared differences between corresponding edges from 1 (or simply, 1 − sum of squared errors).

1 $${{{{R}}}}^2 = 1 - \mathop{\sum}\limits_{i,j}^{{{{N}}}} \left({w_{i \to j}^{{{{{{\mathrm{SZ}}}}}}} - w_{i \to j}^{{{{{{\mathrm{HC}}}}}}}} \right)^2$$

Where *N* is the total number of nodes in the network, *i* and *j* are nodes and $w_{i \to j}$ is an edge between them. Next, we calculated network density *D* or the degree of connectivity within the network, calculated as the fraction of possible edges that are actually present:

2 $${{{{D}}}} = \frac{{\mathop {\sum }\nolimits_{i,j}^{{{{N}}}} w_{i \to j}}}{{{{{{N}}}}({{{{N}}}} - 1)/2}}$$

Where *N* is the total number of nodes in the network, *i* and *j* are nodes and $w_{i \to j}$ is an edge between them. Finally, we visually identified and categorized feedback loops that were identified in each of the models and characterized them as whether they were positive or negative (i.e., leading to increases or decreases in the intensity of affect or behavior), likely adaptive or likely maladaptive (i.e., leading to positive or negative affect), and including context variables or not.

## Data Availability

This clinical data is from a VA study, and it is not publicly available due to privacy concerns, including HIPAA regulations. For access, qualified researchers may contact the corresponding author.

## Code availability

Code used to collect EMA data on smartphones will be made available upon request. Tigramite is a publicly available package available through github (link provided in references). All parameters or customizations to analyze the data have been described in the manuscript in sufficient detail.
